# Erdheim–Chester Disease Due to a Novel Internal Duplication of NRAS: Response to Targeted Therapy with Cobimetinib

**DOI:** 10.3390/ijms242015467

**Published:** 2023-10-23

**Authors:** José A. Riancho, José L. Hernández, Carmen González-Vela, Ana E. López-Sundh, Marcos A. González-Lopez, Francisco Gomez de la Fuente, Remedios Quirce, Eli L. Diamond

**Affiliations:** 1 Servicio de Medicina Interna, Hospital U.M. Valdecilla, Universidad de Cantabria, IDIVAL, CIBERER, 39008 Santander, Spain; joseluis.hernandez@unican.es; 2 Servicio de Anatomía Patológica, Hospital U.M. Valdecilla, Universidad de Cantabria, IDIVAL, 39008 Santander, Spain; 3 Servicio de Dermatología, Hospital U.M. Valdecilla, Universidad de Cantabria, IDIVAL, 39008 Santander, Spain; alopezsundh@gmail.com (A.E.L.-S.); marcosantonio.gonzalez@scsalud.es (M.A.G.-L.); 4 Servicio de Medicina Nuclear, Hospital U.M. Valdecilla, Universidad de Cantabria, IDIVAL, 39008 Santander, Spain; franciscojavier.gomezdela@scsalud.es (F.G.d.l.F.); mremedios.quirce@scsalud.es (R.Q.); 5 Departments of Neurology and Medicine, Memorial Sloan Kettering Center, New York, NY 10065, USA; diamone1@mskcc.org

**Keywords:** histiocytosis, Erdheim–Chester disease, cobimetinib, NRAS, targeted therapy, kinase inhibitors

## Abstract

Histiocytoses encompass a group of exceptionally rare disorders characterized by the abnormal infiltration of tissues by histocytes. Among these, Erdheim–Chester disease (ECD) stands out as a multisystem histiocytosis that typically affects bones and various other tissues. Historically, the treatment of ECD has been challenging. However, recent breakthroughs in our understanding, particularly the discovery of somatic mutations in the RAS-MAPK pathway, have opened new opportunities for targeted therapy in a significant subset of patients with ECD and other histiocytoses. In this report, we present the case of a patient with ECD harboring a previously unidentified microduplication in the NRAS gene in a small fraction of skin cells. This discovery played a pivotal role in tailoring an effective therapeutic approach involving kinase inhibitors downstream of NRAS. This case underscores the crucial role of deep sequencing of tissue samples in ECD, enabling the delivery of personalized targeted therapy to patients.

## 1. Introduction

Histiocytosis comprises a diverse group of disorders characterized by abnormal tissue infiltration of histiocytes and dendritic cells. Previously, it was categorized into two main groups: Langerhans cell histiocytosis and non-Langerhans cell histiocytosis [1]. However, recent classifications have evolved based on molecular insights [2,3] (Table 1). Histiocytoses are rare conditions, with an annual incidence of less than five cases per million [4].

Erdheim–Chester disease (ECD) is a form of non-Langerhans cell histiocytosis with multisystem manifestations. Histologically, it is distinguished by multiorgan infiltration by clonal histiocytes, often characterized by a foamy appearance. Commonly affected tissues include bones, the central nervous system, skin, heart, and peri-renal tissues. The optimal treatment approach remains unclear. Historically, various therapies, including interferon, cladribine, cytokine inhibitors, mTOR inhibitors, and other medications, have been employed with limited success [3,4,5]. Recent discoveries of somatic mutations in the mitogen-activated protein kinase (MAPK) cascade have opened avenues for personalized targeted therapy [6,7].

In this report, we present a case of ECD attributed to a novel NRAS (NRAS Proto-Oncogene, GTPase) mutation that exhibited a remarkable response to cobimetinib, underscoring the significance of molecular diagnostics in tailoring effective targeted therapies.

## 2. Case Report

**Figure 1:**
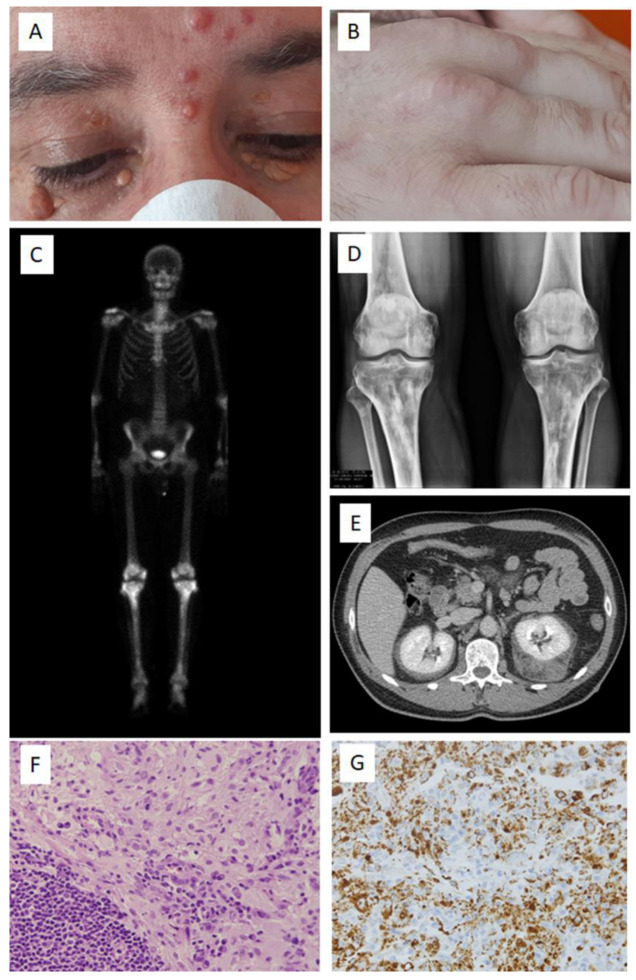
(**A**) Yellowish xanthelasma at the eyelids, as well as several reddish papulo-nodular lesions. (**B**) Tendinous xanthoma over the metacarpal joints. (**C**) Bone scintigraphy showing increased uptake at several metaphyseal regions, more intense in the distal femora and proximal tibia. (**D**) Plain X-ray showing mixed lesions in the femora and tibia. (**E**) Contrast-enhanced CT scan showing a peri-renal infiltration, more marked around the left kidney. (**F**) Biopsy of a nodular skin lesion showing diffuse interstitial accumulation of histiocytes with xanthomatous features and a nodular lymphoplasmacytic infiltrate (×400). (**G**) CD68-positive histiocytes (×200).

A 54-year-old Caucasian man sought evaluation at the outpatient clinic due to multiple skin lesions that had progressed over 15 years. He reported intermittent mild knee pain but was otherwise asymptomatic. His past personal and family medical histories were unremarkable. Physical examination revealed numerous purple cutaneous nodules, predominantly on the face, trunk, and upper limbs. Additionally, palpebral xanthelasma and several tumors were observed over the hands, Achilles tendon, and patellar tendons, consistent with tendinous xanthomas (see Figure 1). A biopsy from one of these lesions, obtained 12 years earlier, was initially diagnosed as “xanthoma”. The rest of the physical examination was unremarkable.

Laboratory tests yielded normal results. Imaging studies revealed peri-renal tissue infiltration and lesions in bone metaphysis, particularly at the femur and tibia (see Figure 1C–E). An FDG-PET scan indicated increased metabolism at cutaneous nodules, peri-renal areas, bones, and certain supradiaphragmatic regions. Cranial MRI and heart ultrasound findings were within the normal range. However, chest MRI demonstrated contrast enhancement at the subendocardial myocardium.

Histopathological examination of a skin nodule biopsy exhibited a mixed infiltrate consisting of foamy histiocytes, plasma cells, lymphocytes, and neutrophils in the dermis (see Figure 1F,G). Most histiocytes were positive for CD68, but negative for CD1A and BRAF. Some were S100-positive, with focal emperipolesis. Plasma cells were IgG-positive, with only scattered IgG4-positive cells. This pattern was diagnostically suggestive of non-Langerhans cell histiocytosis, displaying features akin to juvenile xanthogranuloma and Rosai-Dorfman. Nevertheless, considering the mixed findings, which can also be observed in ECD, and the characteristic systemic disease pattern associated with ECD, a diagnosis of ECD was confirmed.

The patient was initiated on pegylated interferon therapy, but it yielded only a minor reduction in some cutaneous lesions. In pursuit of a more effective treatment strategy, molecular analysis of the tissue sample was conducted through the MSK Make-an-Impact initiative. The specimen was estimated to contain approximately 20% tumor cells. Analysis of 400 target genes revealed an in-frame insertion in exon 3 of NRAS (c. 176–229 dup CTGGACAAGAAGAGTACAGTGCCATGAGAGACCAATACATGAGGACAGGCGAAG), present in 4.9% of the reads. The mutation was absent in peripheral blood cells, and no other pathogenic mutations were identified in either blood or skin samples.

**Figure 2:**
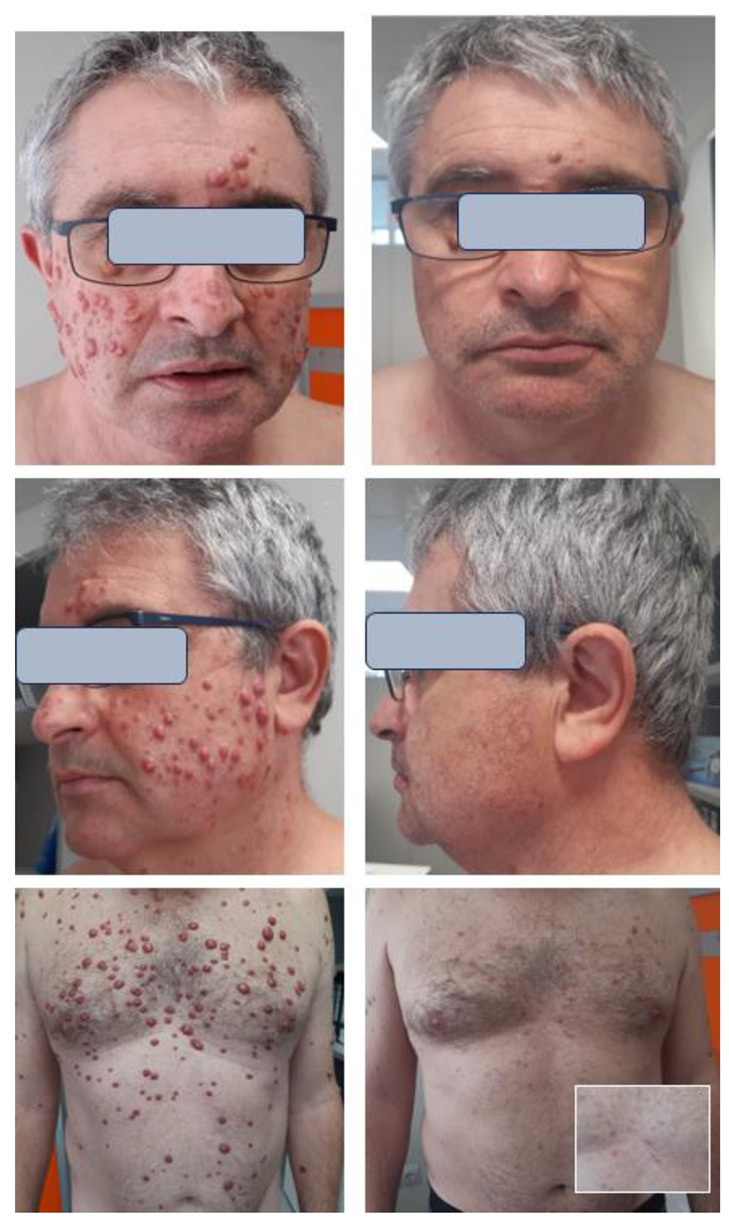
Face and trunk before (**left**) and after 10 months of cobimetinib therapy (**right**). Many papulonodular lesions disappeared, particularly on the face. Most trunk lesions also disappeared or decreased markedly, persisting only a residual non-elevated erythematous macula (see insert at the back). On the contrary, xanthelasma did not show clear changes.

**Figure 3:**
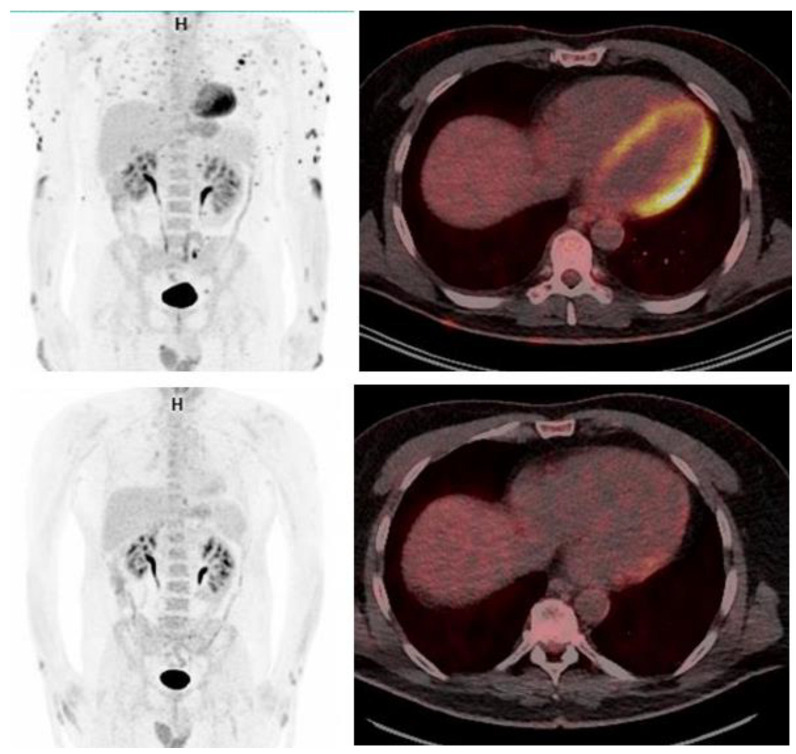
FDG PET scan before (**upper** panels) and after cobimetinib (**lower** panels), showing that most spots of increased metabolic activity, including the heart.

Cobimetinib was initiated at a dose of 40 mg daily. After two weeks, a marked acneiform rash developed on the face and scalp, leading to a temporary discontinuation of the drug. After an additional two weeks, treatment was reintroduced at a daily dose of 20 mg, administered in 21-day cycles every month, without subsequent cutaneous or systemic adverse effects. A prompt response was observed, peaking at around 6–8 months after initiating cobimetinib. Skin nodules either disappeared or markedly regressed (see Figure 2). Visceral lesions also improved, as demonstrated by a clear reduction in peri-renal regions and bone lesions in the FDG PET scan (see Figure 3). However, xanthelasmas and tendinous xanthomas exhibited only a modest decrease in size. The patient’s therapy was discontinued after 10 months. Due to a cutaneous relapse a few months later, cobimetinib therapy was reinitiated and a marked improvement was again observed. 

## 3. Discussion

Approximately 1500 cases of Erdheim–Chester disease (ECD) have been documented since its initial description in 1930 [6]. ECD histiocytes exhibit characteristics typical of non-Langerhans cell histiocytosis, such as CD68+, CD163+, CD1a−, CD207−, and varying levels of S100 expression. However, histological features may sometimes resemble other Group C non-Langerhans cell histiocytosis, such as juvenile xanthogranuloma. Cases with mixed histology, displaying features of both Langerhans cells and Rosai–Dorfman, are not uncommon [4,5,8]. Also, different histiocytoses may show overlapping clinical manifestations and organ involvement [3,4,5]. Among them, multicentric reticulohistiocytosis (MR), which has been included among the C-group (cutaneous) histiocytosis. It may be distinguished from ECD by the presence of numerous multinucleate giant cells and the typical macrophages showing eosinophilic, finely granular, ground glass, cytoplasm, and a virtual absence of foamy cells. MR predominantly affects adult females and involves the skin and the joints, usually as an erosive arthritis [9]. Thus, a definitive diagnosis of ECD relies on consistent clinical and radiological findings, coupled with appropriate histological examination [7]. In the present case, we observed some mixed features in the biopsies. However, given the absence of lymphadenopathy, the characteristic skeletal mixed lesions in the tibiae and femora, and the peri-renal infiltrates (“hairy kidney”), we established the diagnosis of ECD with multisystem involvement. Other disorders that may also mimic ECD did not fit this patient’s phenotype [10]. 

Among the common manifestations of ECD, typical mixed sclerotic/lytic metaphyseal bone lesions are present in approximately 90% of patients, particularly around the knee, and they strongly suggesting ECD. Consequently, the differential diagnosis often includes conditions like osteopoikilosis, tenosynovial giant cell tumors, IgG4-related disease, fibrous dysplasia, and chronic recurrent multifocal osteomyelitis [8]. Other frequent manifestations encompass peri-aortic and peri-renal infiltration (60–65% of cases), occasionally impacting renal function; infiltrative myocardiopathy (30–70%); central diabetes insipidus linked to neurohypophysis infiltration (45%); cerebellar syndrome (20–30%); ocular abnormalities arising from retroorbital infiltration (35%); and skin lesions (20–30%), which may appear either “xanthomatous” or “tumoral” [4,5,7,11].

In recent years, mutational analysis has emerged as a pivotal tool for both diagnosis and personalized therapy of histiocytoses. Somatic activating mutations within the MAPK pathway have been detected in roughly three-quarters of ECD patients. BRAF mutations (present in 50–65% of patients) are the most common, followed by MAP2K1 mutations (found in about 10–20% of patients). These mutations are also prevalent in Langerhans cell histiocytosis, occurring in a similar proportion of patients. In both disorders, the V600E mutation of BRAF is the most frequently observed [5]. Moreover, a recent genome-wide association study suggests that common germinal variants might influence the risk of developing the disease [12]. Genetic variants in the RAS/MAPK/ERK pathway have also been detected in some patients with Rosai–Dorfman disease [13]. 

This report underscores several significant aspects. Firstly, it broadens the mutational spectrum of ECD. NRAS mutations are rare in ECD, with only a few cases reported. Notably, this case represents a novel NRAS mutation, distinct from those found in previous studies [14,15]. Intriguingly, a somewhat similar NRAS internal tandem duplication (albeit shorter; c.164_193dup) was identified in a patient with colon cancer by Nelson et al. [16]. These authors demonstrated that the mutation enhanced the interaction of RAS with RAF (Raf proto-oncogene Ser/Thr kinase), resulting in increased MEK (ERK activator kinase, also known as MAP2K1) phosphorylation and downstream ERK (extracellular signal-regulated kinase) activity. The mutation hindered the interaction with neurofibromin 1 (NF1)-GTPase-activating protein (GAP), thereby sustaining RAS protein activity.

**Figure 4:**
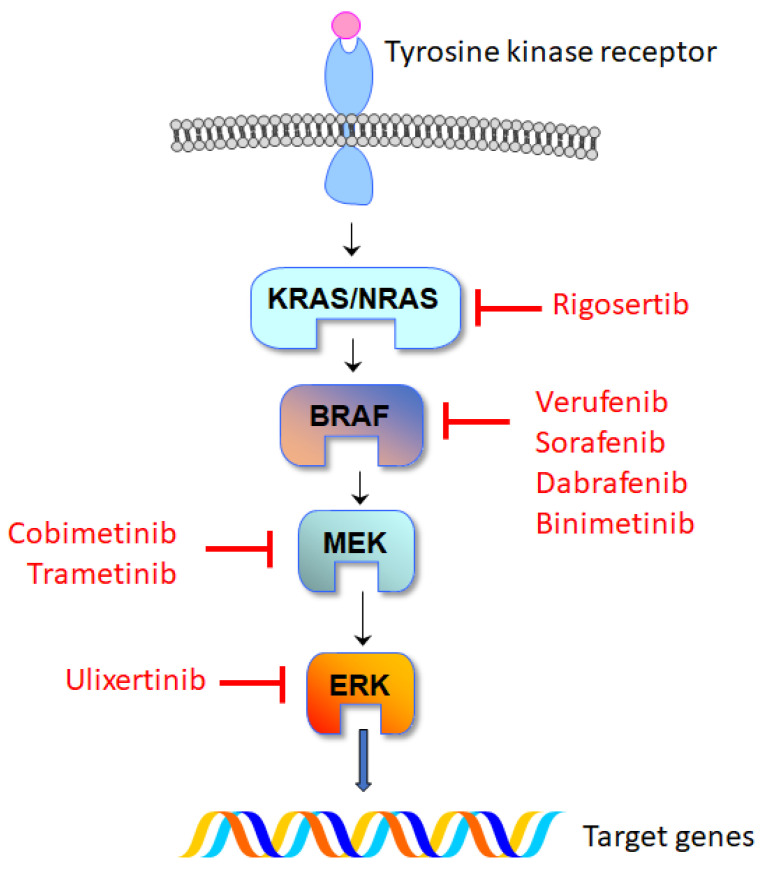
Schematic representation of the BRAF/MAPK pathway and available inhibitors. NRAS: NRAS Proto-Oncogene, GTPase. KRAS: KRAS Proto-Oncogene, GTPase. BRAF: B-Raf Proto-Oncogene, Serine/Threonine Kinase. MEK: MAPK/ERK Kinases (MAP2K, mitogen-activated protein kinase kinases). ERK: extracellular signal-regulated kinases.

Secondly, this case underscores the critical role of expert molecular diagnosis in patients suspected of ECD or other histiocytoses. Vemurafenib is the treatment of choice for patients with BRAF mutations, while MEK inhibitors are recommended in the presence of activating mutations in other MAPK pathway genes. In this case, cobimetinib induced a significant response, aligning with the reported 89% response rate in patients with other MAPK mutations [17]. Cobimetinib’s allosteric inhibition of MEK1/2 reduces ERK1/2 phosphorylation [18]. Apart from vemurafenib and cobimetinib, other small molecules targeting the RAS/MAPK pathway, such as dabrafenib, trametinib, encorafenib, and sorafenib, have been employed, alone or in combination with other drugs, in a small number of patients with L-group histiocytosis [19,20,21] (Figure 4). For patients without druggable mutations or those refractory to kinase inhibitors, treatment options include interferon-α, cladribine, IL-1 inhibitors, glucocorticoids, or chemotherapy [5,22]. In patients with progressive or unresponsive disease, therapy with MEK1/2 inhibitors can be tried, even in the absence of detectable mutations [5,17,18]. Additionally, mTOR inhibitors like sirolimus and everolimus have been used sporadically due to the interactions between RAS/MAPK and mTOR pathways [23]. In some retrospective cohort studies, therapy with either interferon or targeted drugs was associated with better prognosis [24]. This suggests that early therapy must be considered in patients with some signs of progressive disease, even in the absence of significant organ damage. Relapses are common after drug withdrawal [6,7,25], and maintenance therapy is frequently needed. For the rare patients with asymptomatic disease and no impending organ dysfunction, in the absence of clinical trial evidence, a watch and wait approach with close follow-up is a reasonable option [5,26].

This case also underscores the benefits of international collaborations, which enable advanced genotyping for patients worldwide, as previously described in a report that briefly mentioned this case [27].

However, this case raises several questions, including optimal drug dosing and scheduling. Notably, we employed only one-third of the commonly recommended 60 mg dose. Moreover, the maintenance therapy following response induction remains uncertain. Many patients experience disease relapses after discontinuing targeted drug therapy, likely due to the persistence of mutated clones. Intriguingly, some mutation-negative patients may still respond to BRAF/MAPK inhibitors, suggesting potential cryptic mutations in non-sequenced regions or a small, undetectable number of cells. Therefore, it remains unclear whether patients without mutations in molecular analyses are true non-carriers or false negatives. Given the molecular mosaicism in biopsies, deep sequencing procedures are essential to detect mutations expressed in only a small fraction of cells. Other questions warranting further research pertain to the variations in cell biology and pathogenetic mechanisms among various lesion types. As seen in this case, different responses of xanthomatous and nodular cutaneous lesions highlight the need for further elucidation of the involved mechanisms [5].

In summary, we present a case of ECD resulting from a novel NRAS mutation, emphasizing the positive impact of targeted therapy in this disorder.

## Figures and Tables

**Table 1:** Classification of histiocytosis [1,2].

**L Group**
LCH
ICH
ECD
Mixed LCH/ECD
**C Group**
Cutaneous non-LCH ¯ Non-XG family: includes cutaneous RDD ¯ XG family: includes JXG
Cutaneous non-LCH with major systemic component (including MRH)
**R Group**
Sporadic RDD ¯ Classic RDD ¯ Extranodal RDD ¯ RDD with neoplasia or immune disease ¯ Unclassified
Familial RDD
**M Group**
Primary malignant histiocytoses
Secondary malignant histiocytosis (with another haematological malignancy)
**H Group**
Primary HLH (monogenic inherited conditions)
Secondary HLH (non-Mendelian HLH)
HLH of uncertain origin

ECD, Erdheim–Chester disease; HLH, Haemophagocytic lymphohistiocytosis; ICD, indeterminate cell histiocytosis; LCH, Langerhans cell histiocytosis; MRH, multicentric reticulohisticocytosis; RDD, Rosai-Dorfman disease; XG, xanthogranuloma; JXG, juvenile xanthogranuloma.

## Data Availability

Data can be obtained from the authors upon reasonable request.
